# Communicating Hydrocephalus Secondary to Pseudomeningocele following Cervical Spine Surgery: A Case Report

**DOI:** 10.31662/jmaj.2024-0179

**Published:** 2024-11-01

**Authors:** Yushi Sakamoto, Nobuaki Taniguchi, Kosuke Iwaisako

**Affiliations:** 1 Department of Spine Surgery, Yonemori Hospital, Kagoshima, Japan; 2 Department of Neurosurgery, Yonemori Hospital, Kagoshima, Japan

**Keywords:** communicating hydrocephalus, pseudomeningocele, cerebrospinal fluid, ventriculoperitoneal shunt

## Abstract

Pseudomeningocele (PMC) after spinal surgery involves cerebrospinal fluid (CSF) continuously leaking from a compromised dura mater and accumulating subcutaneously. PMC is a rare postcervical spine surgery that can be spontaneously resolved; therefore, asymptomatic cases are often observed. This report presents a case of communicating hydrocephalus resulting from PMC following posterior decompression at the craniocervical junction. A 77-year-old man with advanced dementia and a history of C2-T3 posterior fixation was admitted after a head injury, presenting quadriplegia at the MMT2 level. Magnetic resonance imaging (MRI) revealed severe spinal cord compression at C1/2. A posterior decompression of the craniocervical junction was performed. However, dura mater damage occurred during surgery, and the damaged area was repaired with artificial dura mater and fibrin glue. One month postsurgery, subcutaneous swelling was observed and an MRI identified PMC. As the patient was asymptomatic, observation was chosen. Four months postsurgery, the patient exhibited drowsiness and vomiting. An MRI was conducted, revealing the presence of communicating hydrocephalus. Ventricular drainage and ventriculoperitoneal shunt were performed, and the hydrocephalus and PMC improved. Dural injury during spinal surgery is a relatively common complication; but, if inadequately repaired, CSF leakage may persist and lead to PMC. Persistent leakage of CSF into the PMC may have hindered CSF absorption, leading to communicating hydrocephalus. Severe cognitive impairment and quadriplegia may have hindered neurological evaluation and delayed the detection of the hydrocephalus, and more careful follow-up would have been desirable.

## Introduction

Pseudomeningocele (PMC) following spinal surgery is a condition where cerebrospinal fluid (CSF) continuously leaks from the compromised dura mater and subcutaneously accumulates ^(1)^. PMC is an uncommon complication of cervical spine surgery that may be spontaneously resolved over time; thus, asymptomatic cases are often observed ^(2)^. If PMC persists for a long time, it can lead to hydrocephalus in the chronic phase, particularly in the lumbar spine ^(3), (4)^. Here, we present a case of communicating hydrocephalus, resulting from PMC following posterior decompression at the craniocervical junction.

## Case Report

**Figure 1.**
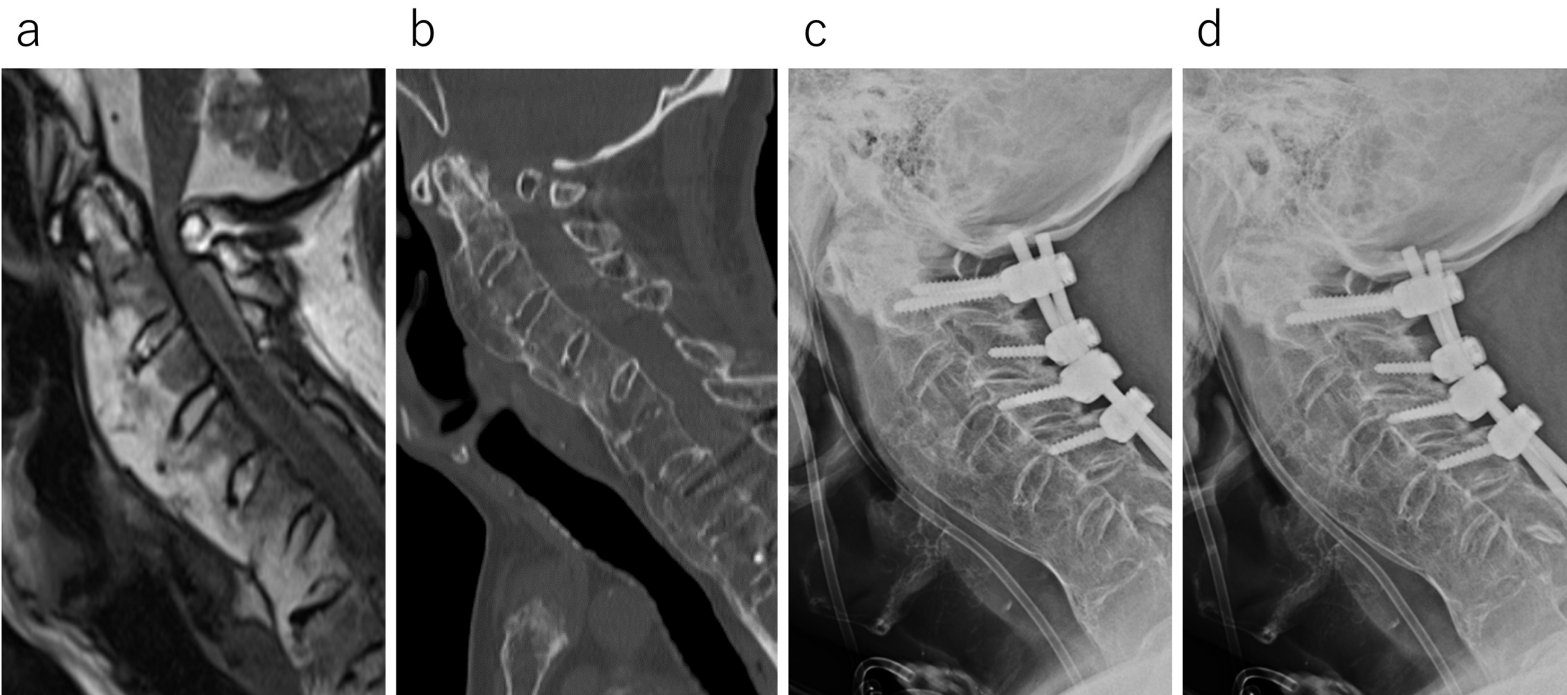
Imaging tests are recommended for the initial consultation. Sagittal T2-weighted MRI demonstrates substantial spinal cord compression at C1/2 (a). The atlantodental interval measures approximately 1 mm on the sagittal CT scan (b). X-ray images of anteflexion (c) and retroflexion (d) similarly reveal minimal instability at C1/2.

A 77-year-old male with advanced dementia and a history of C2-T3 posterior fixation was admitted to the emergency room following a head injury. Quadriplegia was observed, with muscle strength at the MMT2 level, and magnetic resonance imaging (MRI) revealed a retro-odontoid pseudotumor and severe spinal cord compression by the posterior arch at the C1 level (Figure 1a). Sagittal CT showed that the atlantodental interval was approximately 1 mm (Figure 1b), and even when viewing the anteroposterior bending and retroflexion X-ray images (Figure 1c and d), almost no instability was observed between C1 and C2. Consequently, posterior decompression of the craniocervical junction was performed. During partial occipital resection, the dura mater was damaged and subsequently repaired using artificial dura mater and fibrin glue. To ensure the integrity of the repair, the Valsalva maneuver was performed, confirming the absence of CSF leakage.

**Figure 2.**
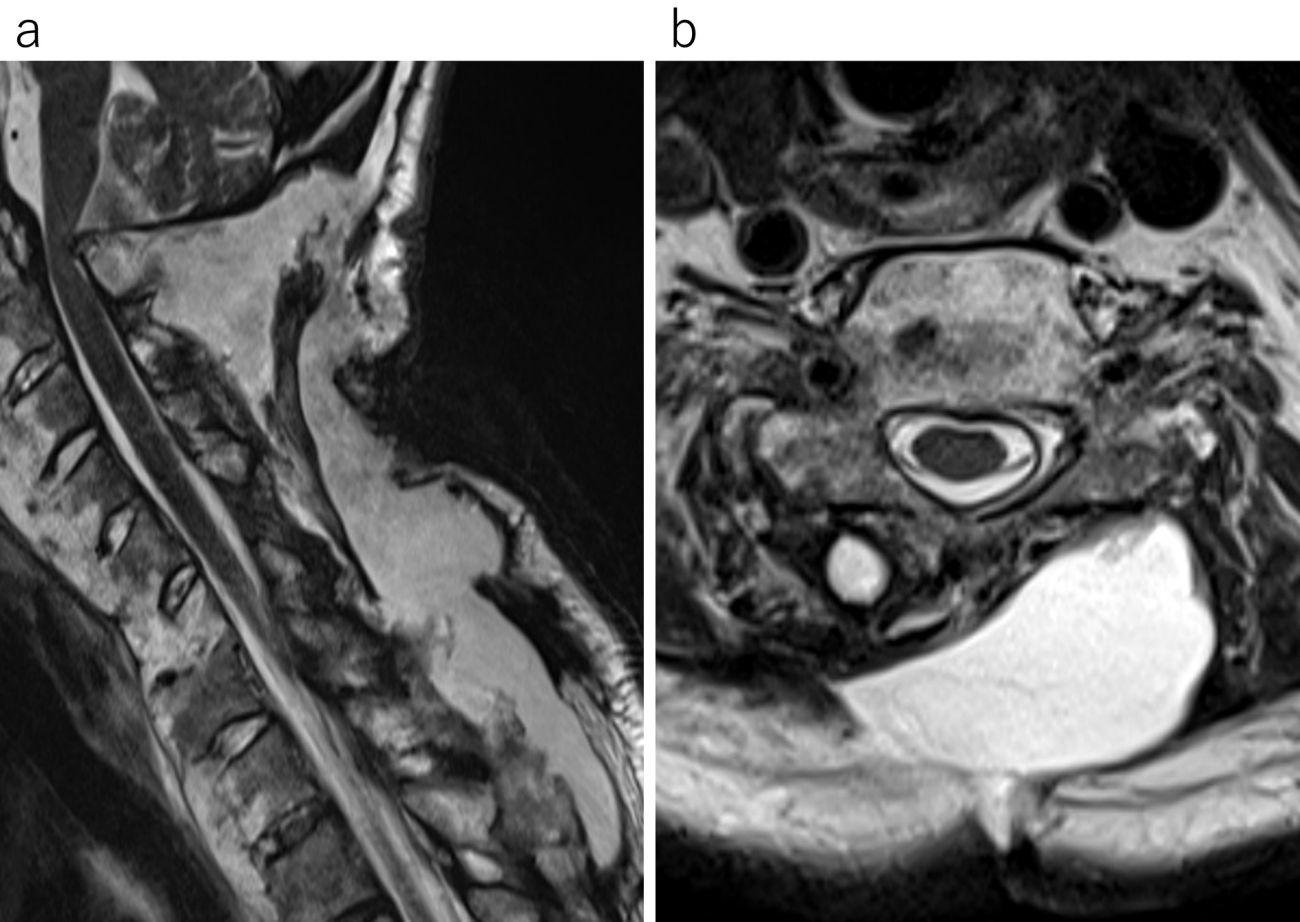
Approximately 1 month postsurgery, subcutaneous swelling was noted, prompting an MRI evaluation. The imaging revealed a pseudomeningocele measuring 14 cm in length on the sagittal plane (a) and 4 cm in width on the horizontal plane (b).

**Figure 3.**
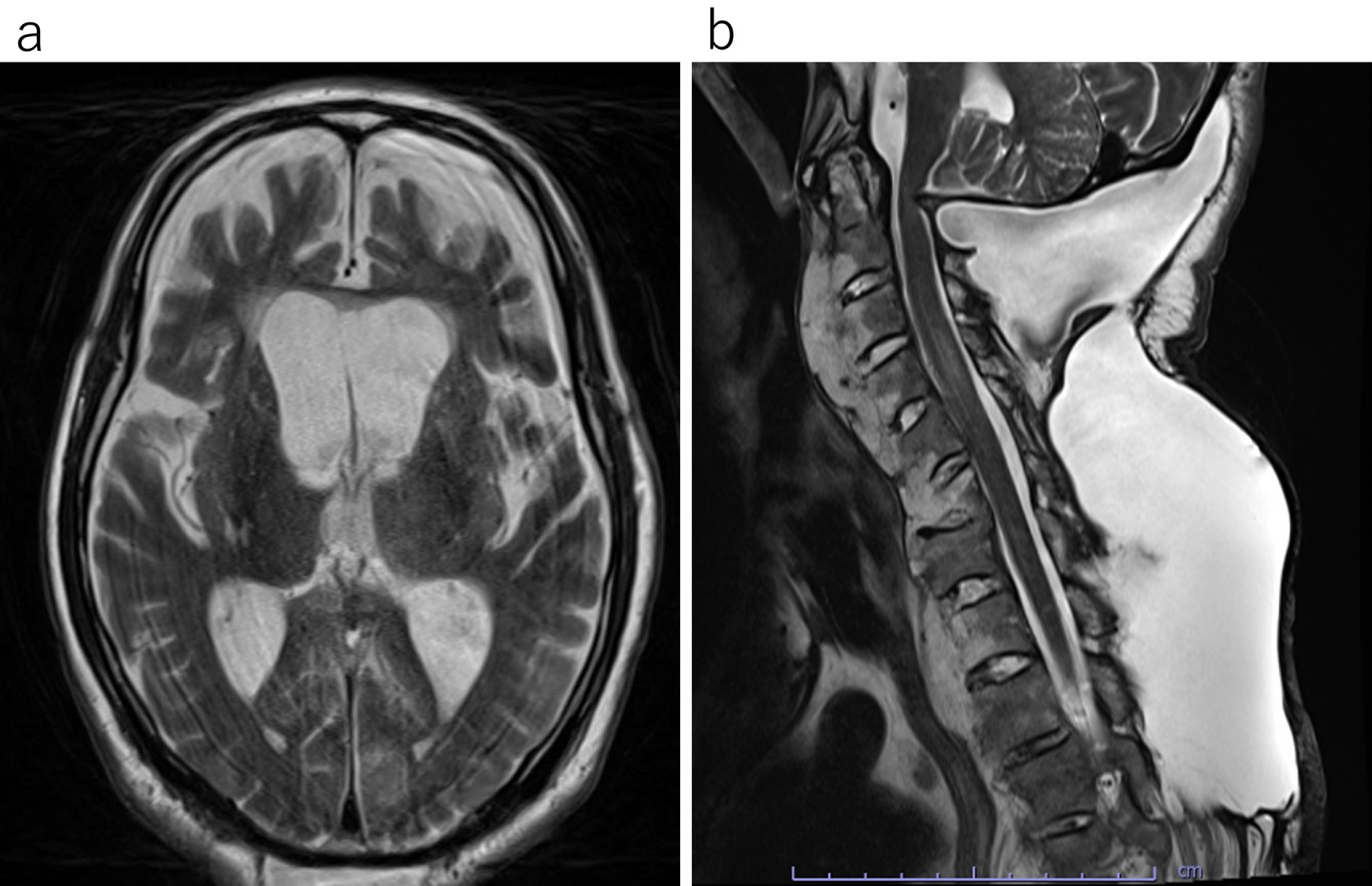
Four months postsurgery, the patient exhibited drowsiness and vomiting. Head and cervical MRIs were performed in response to these symptoms. The head MRI revealed considerable ventricular enlargement (a), whereas the cervical MRI indicated an increase in the pseudomeningocele (b).

**Figure 4.**
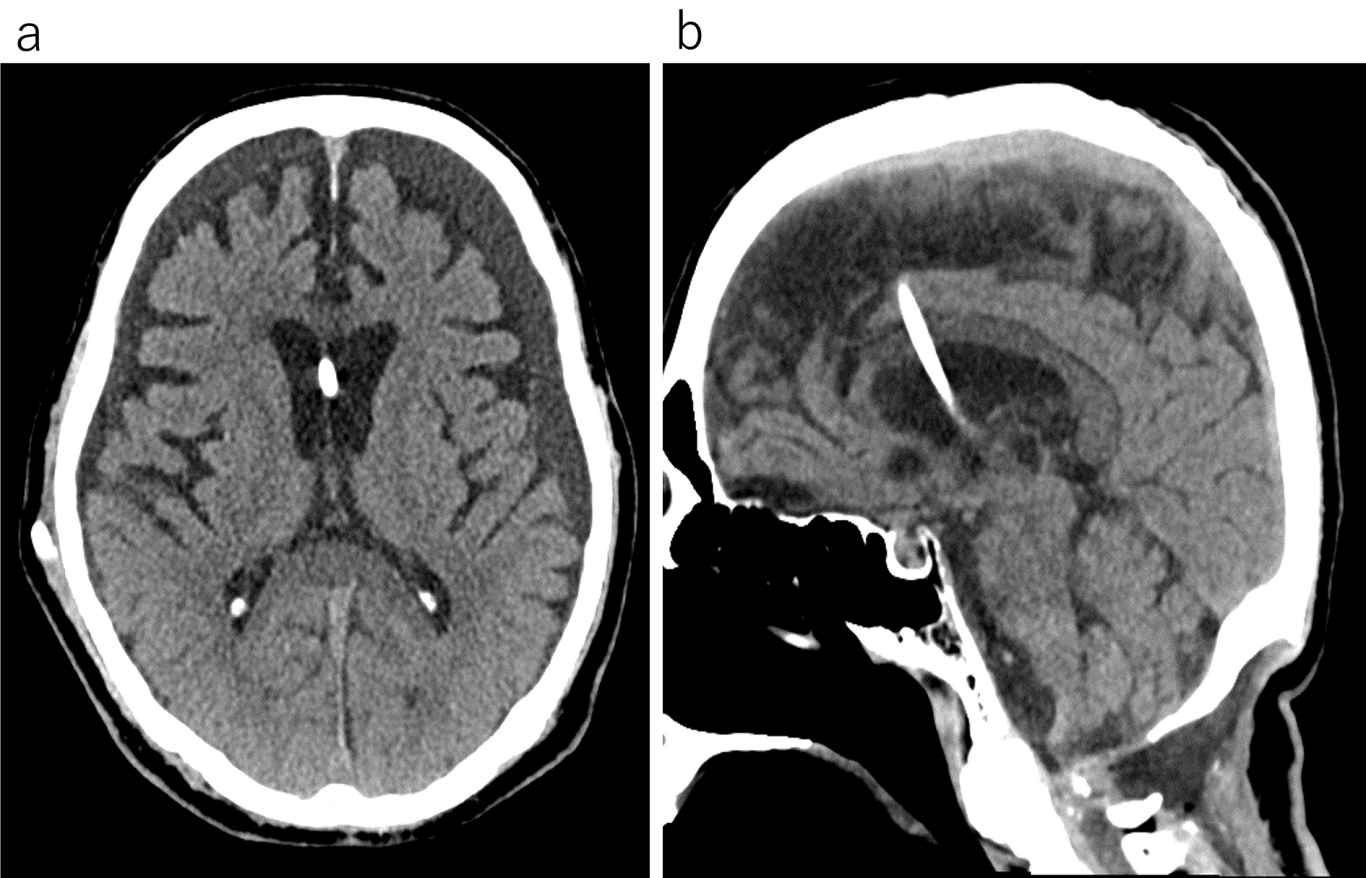
This computed tomography scan of the head, performed after ventriculoperitoneal shunt surgery, shows that the ventricles have returned to normal size (a) and the pseudomeningocele has significantly decreased (b).

Postoperative rehabilitation commenced. However, subcutaneous swelling was noted and an MRI indicated PMC one month postsurgery (Figure 2). As no significant symptoms were present at that time, rehabilitation continued with careful monitoring. Approximately 4 months postsurgery, the patient experienced several days of daytime drowsiness and vomiting. An MRI revealed considerable ventricular enlargement, leading to a diagnosis of communicating hydrocephalus (Figure 3), and immediate ventricular drainage was performed. At the time of drainage, the intracranial pressure was 8 cm H_2_O. CSF examination showed a cell count of 2/μL and CSF protein of 43 mg/dL. A ventriculoperitoneal (VP) shunt was implanted one week later, resulting in an improvement of the hydrocephalus and PMC (Figure 4), and the patient resumed gait training.

## Discussion

Dural injury during spinal surgery is a common complication ^(5)^, but PMC occurs when CSF leakage persists and accumulates in the subcutaneous space. Ailon et al. reported that the incidence of PMC after cervical spine surgery is approximately 0.08%, with 77% of these cases occurring during posterior surgery ^(2)^. However, the exact incidence is unknown, as small PMCs may go undetected. Asymptomatic cases are usually closely monitored, as PMC may spontaneously resolve ^(6)^.

When the fistula between the subarachnoid space and the leakage site closes, stopping the inflow of CSF, the CSF within the PMC is absorbed by capillaries and eventually dissipates. Herein, the fistula remained open, leading to a sustained decrease in cerebrospinal pressure. The decrease inhibited CSF absorption into the arachnoid granulations and caused a compensatory increase in CSF production. However, owing to the limited elasticity of the skin, the growth of the PMC eventually slows. CSF production is speculated to surpass the amount entering the PMC and the amount that can be absorbed by the arachnoid granulations, leading to communicating hydrocephalus. Herein, the condition required urgent treatment and ventricular drainage and VP shunt surgery were promptly performed. Therefore, not only did the hydrocephalus improve, but the PMC also resolved.

Whether to perform CSF leak repair or drainage first is difficult to decide. However, in cases such as this one where consciousness is impaired, we believe drainage must be prioritized. Reports have been available of patients who underwent dural repair for PMC and the hydrocephalus following lumbar spine surgery; however, the hydrocephalus did not improve, and VP shunt surgery was required ^(7), (8)^. If impaired absorption of CSF occurs, securing an outflow route for CSF using a VP shunt procedure or another method is necessary, as simply closing the damaged area of the dural membrane is insufficient.

This case was complicated by severe cognitive impairment and quadriplegia, hindering effective communication for a thorough neurological evaluation. If objective neurological findings had been meticulously assessed and imaging tests had been conducted, repairing the dural damage before the onset of hydrocephalus might have been possible. Additionally, the persistence and growth of PMC following spinal surgery may serve as a precursor to communicating hydrocephalus, thereby necessitating careful and continuous follow-up.

## Article Information

### Conflicts of Interest

None

### Author Contributions

Yushi Sakamoto: manuscript writing, design, and editing.

Nobuaki Taniguchi and Kosuke Iwaisako: care of patient and guiding manuscript writing.

### Informed Consent

The patient has understood and consented to the anonymous submission of the case report to a medical journal.
